# Akutes Kompartmentsyndrom der Extremitäten

**DOI:** 10.1007/s00104-022-01624-9

**Published:** 2022-03-29

**Authors:** Y. Kalbas, Y. Kumabe, R. M. Sellei, H. C. Pape

**Affiliations:** 1 Klinik für Traumatologie, UniversitätsSpital Zürich, Rämistrasse 100, 8091 Zürich, Schweiz; 2 Klinik für Unfallchirurgie und orthopädische Chirurgie, Sana Klinikum Offenbach, Starkenburgring 66, 63069 Offenbach, Deutschland

**Keywords:** Kompartmentsyndrom, Weichteilschaden, Ischämie, Perfusion, Fasziotomie, Compartment syndrome, Soft tissue injury, Ischemia, Perfusion, Fasciotomy

## Abstract

Das akute Kompartmentsyndrom der Extremitäten ist ein chirurgischer Notfall, dessen schnelle Diagnose und sofortige operative Therapie essenziell für das Outcome sind. Ursächlich ist ein Anstieg des Gewebedruckes innerhalb einer durch Faszien eingeschlossenen Muskelloge und eine daraus resultierende Mikroperfusionsstörung. Diese kann potenziell desaströse Folgen, wie den Verlust der Extremität durch großflächige Nekrosen oder eine vitale Bedrohung durch infektiöse Komplikationen, haben. Obwohl meist traumatisch bedingt, kann auch eine Vielzahl anderer Ursachen zur Entstehung eines Kompartmentsyndroms führen, sodass eine Grundkenntnis dieses Zustandsbildes nicht nur für Unfallchirurgen von großer Wichtigkeit ist. Dies gilt vor allem, weil eine zeitige Therapie eine schnelle Diagnose- und Indikationsstellung erfordert. Im folgenden Artikel wird ein Überblick über die zugrunde liegende Pathophysiologie, die Ursachen, die Symptome sowie die Therapie des akuten Kompartmentsyndroms dargestellt.

## Abstract (en)

Acute compartment syndrome of the extremities is a surgical emergency and a rapid diagnosis and immediate surgical treatment are essential for the outcome. The cause is an increase in the tissue pressure inside a muscle compartment enclosed by fasciae and the resulting disruption of microperfusion. This can have potentially disastrous consequences, such as loss of the extremity due to extensive tissue necrosis or a threat to life due to infectious complications. Although mostly triggered by trauma, a multitude of other causes can lead to the formation of a compartment syndrome, so that a basic knowledge of this condition is of great importance not only for trauma surgeons. This is particularly true because a timely treatment necessitates rapid diagnosis and evaluation of the indications. This article provides an overview of the underlying pathophysiology, the causes, the symptoms and the treatment of acute compartment syndrome.

## Lernziele

Nach der Lektüre dieses Beitrags …

- kennen Sie die Pathophysiologie, die Ätiologie und die Epidemiologie des akuten Kompartmentsyndroms der Extremitäten,
- erkennen Sie die klinischen Zeichen eines akuten Kompartmentsyndroms und kennen mögliche diagnostische Hilfsmittel,
- können Sie die Indikation zur Dermatofasziotomie stellen und kennen deren Durchführung und Nachbehandlung.

## Hintergrund

Die Erstbeschreibung des Kompartmentsyndroms bzw. der daraus resultierenden ischämischen Kontrakturen erfolgte bereits 1881 durch den deutschen Chirurgen **Richard von Volkmann**

Richard von Volkmann

[1]. 1958 bis 1966 wurde dieses Krankheitsbild durch **Ellis**

Ellis

und **Seddon**

Seddon

insbesondere im Zusammenhang mit Unterschenkelfrakturen beschrieben und die zeitige **operative Faszienspaltung**

operative Faszienspaltung

als Therapie propagiert [2, 3]. Seitdem sind weitere wichtige Arbeiten zu dem Krankheitsbild erschienen, so unter anderem auch 1975 eine Arbeit von **Matsen**

Matsen,

die den Begriff **Kompartmentsyndrom**

Kompartmentsyndrom

prägte und als einen „Zustand, in dem Zirkulation und Funktion in einem anatomischen geschlossenen Raum durch einen Anstieg des inneren Gewebedruckes eingeschränkt werden“ definierte [4].

## Pathophysiologie

Als Kompartiment bzw. Muskelloge bezeichnet man einen von starrer Faszie umgebenen vordefinierten anatomischen Raum.

Beim Erwachsenen beträgt der **Gewebedruck**

Gewebedruck

innerhalb einer Muskelloge etwa 0–4 mm Hg in Ruhe und bis zu 8–10 mm Hg während Belastung [5]. Wenn sich das Volumen innerhalb einer Muskelloge erhöht (z. B. durch eine traumatisches Ödem) oder die Loge eingeengt wird, steigt mit dem Gewebedruck auch der **postkapillare Venendruck**

postkapillare Venendruck

und die lokale Mikrozirkulation wird eingeschränkt [6].

Steigt der Druck über einen gewissen Schwellenwert, so führt die **verminderte Gewebeperfusion**

verminderte Gewebeperfusion

zur ischämischen Zellschädigung: Diese entsteht durch eine Kombination aus Hypoxie, oxidativem Stress und intrazellulärem Ödem [7]. Die resultierende **osmotische Dysbalance**

osmotische Dysbalance

verstärkt das intrazelluläre Odem und führt zur schließlich zur Nekrose. Dies wiederum führt zur lokalen Freisetzung von Entzündungsmediatoren, die abermals das interstitielle Ödem verstärken und den Kompartmentdruck erhöhen [8].

**Figure 1:**
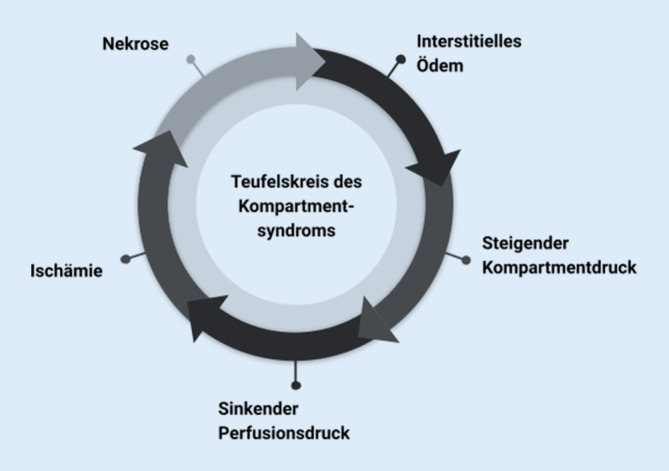

Das Ergebnis ist ein Teufelskreis aus sich gegenseitig intensivierenden Prozessen, die innerhalb kürzester Zeit zum permanenten Funktionsverlust eskalieren können (Abb. 1):

Hierbei ist der zeitliche Verlauf abhängig von dem **Gewebetyp**

Gewebetyp:

Während **Nervenzellen**

Nervenzellen

nach 60 min mit einer Nervenleitungsstörung reagieren und bereits nach 4 h irreversibel geschädigt sein können, tolerieren **Muskelzellen**

Muskelzellen

eine Ischämie bis zu 3–4 h ohne bleibende Schäden [9]. Nach einer Ischämiezeit von 6–12 h ist mit großer Wahrscheinlichkeit mit dauerhaften lokalen Komplikationen wie großflächigen Nekrosen, Infektionen und ischämischen Muskelkontrakturen zu rechnen [10, 11]. Neben solchen lokalen Komplikationen gilt es allerdings auch, potenziell tödliche, systemische Folgen zu bedenken: Hierzu zählen vor allem die **akute Nierenschädigung**

akute Nierenschädigung

als Folge der Rhabdomyolyse, das **Ischämie-Reperfusions-Syndrom**

Ischämie-Reperfusions-Syndrom

durch Elektroytentgleisungen, das „systemic inflammatory response syndrome“ durch übermäßige Zytokinfreisetzung und septische Komplikationen einer lokalen Infektion.

## Ursachen

Dem akuten Kompartmentsyndrom an den Extremitäten liegen zumeist **stumpfe Traumata**

stumpfe Traumata

mit Frakturen zugrunde [12]. In bis zu 30 % der Fälle wird allerdings auch ein Kompartmentsyndrom ohne das Vorliegen einer Fraktur beschrieben [12]: Hierbei werden neben ausgedehnten Weichteilverletzungen ohne Knochenbruch auch (zirkuläre) **thermische Schädigungen**

thermische Schädigungen

und Stromverletzungen, temporäre vaskuläre Okklusionen oder Gefäßverletzungen, raumfordernde Prozesse wie Blutungen [13] oder Tumoren und iatrogene Ursachen wie zu enge Gipse/Verbände oder **Lagerungsschäden**

Lagerungsschäden

beschrieben [14]. Weitere Ursachen können Überanstrengung, nephrotisches Syndrom oder bakterielle Infektionen sein [15]. Eine Übersicht über mögliche Ursachen wird in Tab. 1 dargestellt.

**Table 1:** 

	Traumatisch	Iatrogen	Sonstige
*Volumenzunahme innerhalb des Kompartments*	Frakturen (geschlossen/offen) Ausgedehntes Weichteiltrauma (Gefäßverletzungen) Einblutung, Ödem	Fehlinfusionen	Reperfusion nach vaskulärer Okklusion Atraumatische Blutungen Tumoren Überanstrengung Nephrotisches Syndrom Schlangenbiss Infektionen
*Volumenabnahme des Kompartments*	Thermische Schäden/Stromverletzung	Einengende Gipse/Verbände Chirurgischer Faszienverschluss Extensionsbehandlung Lagerungsschäden	–

## Epidemiologie

Die Inzidenz des akuten Kompartmentsyndroms der Extremitäten wurde durch **McQueen**

McQueen

in einer wichtigen Studie mit 7,3/100.000 bei männlichen und mit 0,7/100.000 bei weiblichen Erwachsenen beschrieben. Zudem wurde gezeigt, dass die Inzidenz besonders hoch (3- bis 30-fach höher) für Patienten unter 35 Jahren ist [12]. Dieselbe Studie beschreibt die **Tibiaschaftfraktur**

Tibiaschaftfraktur

mit 36 % der Fälle als häufigste Ursache. Hiervon waren 83 % der Frakturen geschlossen [12]. Ebenfalls häufig mit Kompartmentsyndrom assoziiert sind Frakturen des Vorderdarms, insbesondere Schaftfrakturen [12]. Neben der Lokalisation wird auch der Unfallmechanismus (**Hochrasanztrauma**

Hochrasanztrauma)

als Risikofaktor für das akute Kompartmentsyndrom beschrieben [16, 17]. Hierbei ist dann ebenfalls der Fuß als eine häufig betroffene Region zu nennen, insbesondere nach Luxationsverletzungen [18, 19].

### Merke

*Studienlage:* Die o. g. Inzidenzen sind mit einem gewissen Vorbehalt zu betrachten, da sie relativ alt sind und es nur wenige aktuelle epidemiologische oder Registerstudien gibt. Daten aus aktuelleren Studien über isolierte Körperregionen scheinen aber bezüglich Alter und Geschlecht übereinzustimmen [17].

### Merke

*Risikopatient:* Am häufigsten betroffen sind junge Männer mit Frakturen von Tibiaschaft und Vorderarm [12]. Das Risiko und auch die Komplikationsrate ist bei Hochrasanztraumata am höchsten [20].

### Cave

*Offene Fraktur:* Eine offene Fraktur macht ein Kompartmentsyndrom zwar weniger wahrscheinlich, aber schließt es nicht aus [21].

## Diagnostik

Die Diagnosestellung des akuten Kompartmentsyndroms beim bewusstseinsklaren Patienten sollte sich vorwiegend auf die klinische Untersuchung stützen [22]. Hierbei ist ein unverhältnismäßiger **therapieresistenter Schmerz**

therapieresistenter Schmerz

als das wichtigste Symptom zu werten:

- „pain out of proportion“ (unverhältnismäßiger Schmerzen),
- „pressure“/„palpably tense compartment“ (Druck/gespanntes Kompartiment),
- „pain with passive stretch“ (Schmerzen bei passiver Dehnung)
- „pallor“ (Blässe),
- „paresthesia“ (Missempfindung),
- „paralysis“ (motorische Schwäche der Kennmuskeln),
- „poikilothermia“ (Kältegefühl).

### Cave

*Pulslosigkeit:* Pulslosigkeit ist kein erforderliches Kriterium für das akute Kompartmentsyndrom: Auch Kompartmentdrücke weit unterhalb der Systole reichen aus, um die Gewebeperfusion vollständig zu unterbinden.

Bei Patienten mit einer eingeschränkten Fähigkeit sich mitzuteilen oder einem gestörten Schmerzempfinden ist die klinische Untersuchung allerdings deutlich erschwert. Dies gilt z. B. für Kleinkinder oder **alkoholisierte Patienten**

alkoholisierte Patienten

sowie für Patienten mit schweren Begleitverletzungen, Neuropathien, Plegien oder Patienten nach Regionalanästhesie. Noch schwieriger kann die Diagnosestellung bei **sedierten Patienten**

sedierten Patienten

auf der Intensivstation sein. Hier gilt es auch, auf indirekte Schmerzzeichen wie z. B. einen Anstieg des Pulses, des Blutdrucks oder des Sedationsbedarfes zu achten.

## Logendruckmessung

Gelingt es anhand der klinischen Untersuchung nicht, eine eindeutige Diagnose zu stellen, sollte eine **diagnostische Logendruckmessung**

diagnostische Logendruckmessung

in Betracht gezogen werden.

## Laborparameter

Für die Diagnose des akuten Kompartmentsyndrom gibt es **keine spezifischen Laborparameter**

keine spezifischen Laborparameter.

Muskelmarker wie Kreatinkinase und Myoglobin sollten aufgrund ihrer fehlenden Spezifität nicht isoliert betrachtet werden [8].

### Cave

*Diagnostik: *Unterstützende Diagnostika können zwar bei unklarer klinischer Untersuchung zur Entscheidungsfindung beitragen, sollten aber unter keinen Umständen bei eindeutiger Diagnose verwendet werden, da hierdurch ein Zeitverlust bis zur operativen Therapie riskiert wird.

## Drohendes Kompartmentsyndrom

Bei einem Patienten mit suggestiver, aber noch nicht eindeutiger Klinik und passendem Unfallmechanismus muss bedacht werden, dass die Entwicklung eines Kompartmentsyndroms ein schneller und **dynamischer Prozess**

dynamischer Prozess

ist. Hier gilt es, einen weiteren Druckanstieg innerhalb der Muskellogen zu vermindern, indem einengende Gipse oder Verbände umgehend entfernt werden und die Extremität auf Herzniveau gelagert wird.

Gleichzeitig ist es ratsam, das Operationsteam und die Anästhesie über das drohende Kompartmentsyndrom zu informieren und die nötige Infrastruktur für einen Notfalleingriff sicherzustellen. **Regelmäßige Reevaluationen**

Regelmäßige Reevaluationen

sind unumgänglich!

### Merke

*Lagerung:* Beim Anheben über das Herzniveau kann die Perfusion verringert werden, die Lagerung unter Herzniveau kann den venösen Rückfluss vermindern und so den Druck weiter steigern.

### Cave

*Kunstfehler:* Ein verpasstes Kompartmentsyndrom gilt als Kunstfehler.

### Merke

*Medikamentöse Therapie:* Für das drohende Kompartmentsyndrom wird ein vorteilhafter Effekt von Antikoagulanzien oder Steroide beschrieben. Bei einem manifesten Kompartmentsyndrom kann die Gewebeperfusion nur durch die operative Therapie wieder sichergestellt werden.

## Therapie

Das einzige operative Verfahren zur Therapie des akuten Kompartmentsyndrom ist die **Dermatofasziotomie**

Dermatofasziotomie

[23]. Die Indikation ist anhand der klinischen Untersuchung sowie der unterstützenden apparativen Diagnostik zu stellen. Weitere Kriterien, die beachtet werden sollten, sind das **Verletzungsmuster**

Verletzungsmuster

(Hochrasanztraumata mit ausgedehnter Weichteilverletzung oder längerer Ischämiezeit z. B. bei Gefäßverletzungen). Beim Polytrauma mit Begleitverletzungen und hämodynamischer Instabilität sollte die Indikation großzügiger gestellt werden.

## Durchführung der Dermatofasziotomie

Ziel der Dermatofasziotomie ist es, sämtliche Kompartimente der betroffenen Extremität vollständig longitudinal zu spalten. Hierfür sollten großzügige **erweiterbare Längsinzisionen**

erweiterbare Längsinzisionen

der Haut gewählt werden, die eine ausreichende Exposition der darunterliegenden Muskellogen bieten. Die grundlegenden Prinzipien der Chirurgie, insbesondere bezüglich der minimalen Abstände zwischen Inzisionen, sollten dabei unbedingt eingehalten werden. Aus diesem Grund ist es zu empfehlen, die Schnittführung über den **intermuskulären Septen**

intermuskulären Septen

zu wählen, um das Spalten mehrerer Kompartimente über einen Zugang zu ermöglichen. Zur Vorbeugung infektiöser Komplikationen ist es essenziell, die **Vitalität der Muskulatur**

Vitalität der Muskulatur

zu überprüfen und nekrotisches Gewebe zu débridieren. Sowohl die Faszien als auch die Haut müssen offen belassen werden, um eine erneute Kompression der Kompartimente zu vermeiden. Da die Wunden steril bedeckt und die Wundränder vor Retraktion geschützt werden müssen, eignen sich zum temporären Wundverschluss die Versiegelung mittels **Vakuumverband**

Vakuumverband

oder die Kombination aus Dermatotraktion mit sterilen Verbänden. Ein **„second look“**

„second look“

mit einer Reevaluation der Muskulatur und einem (partiellen) Wundverschluss sollte innerhalb von 48 h durchgeführt werden. Hierbei ist es wichtig zu beachten, dass die Faszien nicht mehr sekundär verschlossen werden.

### Merke

*Begleitende Frakturen:* Durch das Eröffnen der Muskellogen werden insbesondere Schaftfrakturen der langen Röhrenknochen weiter destabilisiert und sollten dementsprechend unbedingt operativ gesichert werden. Temporäre Stabilisation mit einem externen Fixateur oder intramedullärer Nagelung sind hierbei der Plattenosteosynthese aufgrund der Weichteilproblematik und dem Infektionsrisiko vorzuziehen. Bei gelenknahen Frakturen, die im weiteren Verlauf eine Plattenosteosynthese benötigen, ist es wichtig, die Schnittführung der Dermatofasziotomie entsprechend zu planen. Bei der definitiven Versorgung lassen sich die Schnitte dann zum optimalen Zugang erweitern.

Für die Durchführung einer Dermatofasziotomie bedarf es einer präzisen **Kenntnis der Muskellogen**

Kenntnis der Muskellogen

und deren räumlichen Lagebeziehungen zueinander sowie zu den **neurovaskulären Strukturen**

neurovaskulären Strukturen.

Wir stellen hier eine geläufige Herangehensweise am Unterschenkel als Beispiel dar:

Um alle 4 Kompartimente des **Unterschenkels**

**Figure 2:**
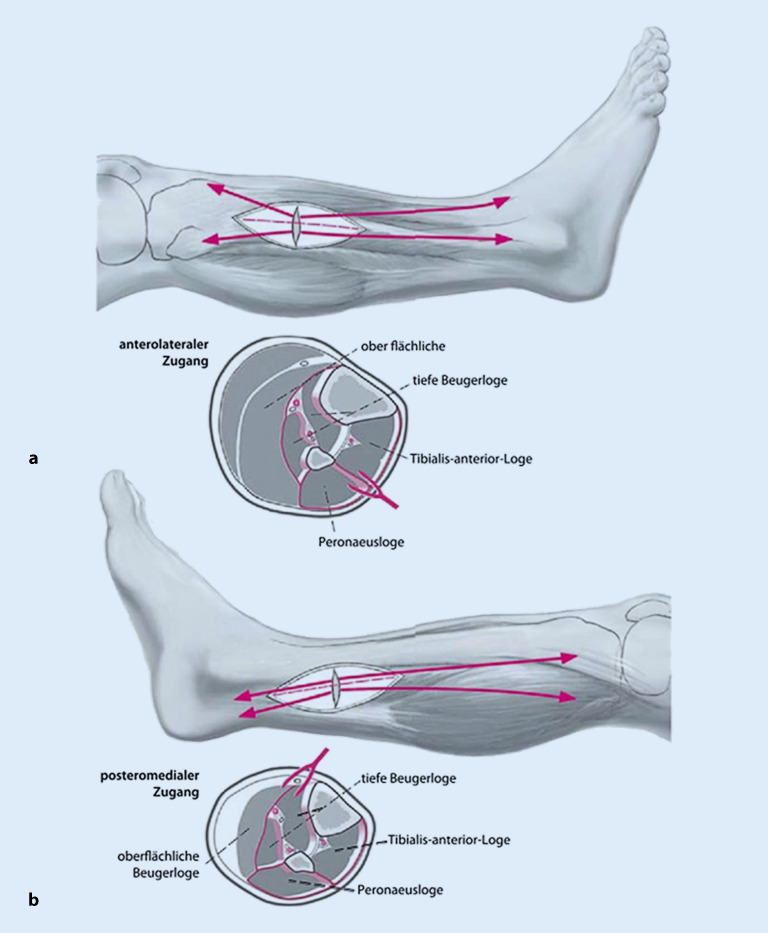

**Figure 3:**
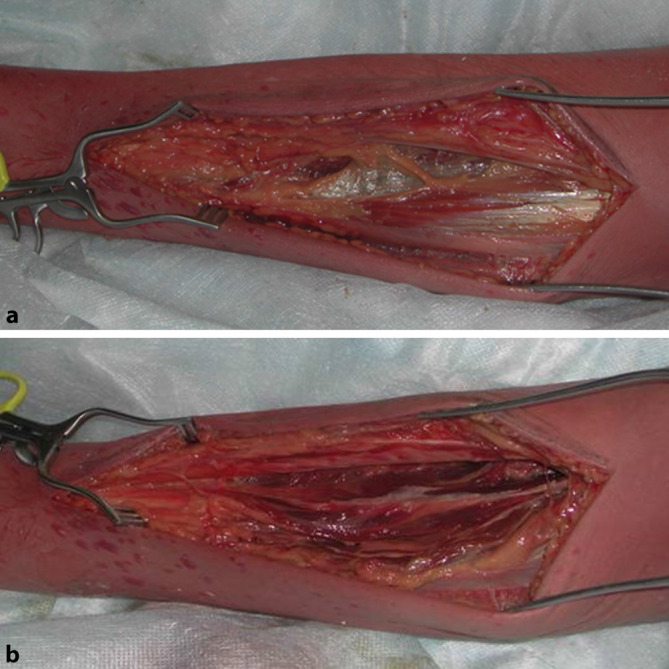

Unterschenkels

ausreichend darstellen und eröffnen zu können ist ein **bilateraler Zugang**

bilateraler Zugang

eine gute Möglichkeit (Abb. 2; [24]). Die **medialseitige Inzision**

medialseitige Inzision

ist ca. 2 cm unterhalb der Tibiahinterkante zu wählen. Hierbei gilt es, insbesondere den N. saphenus und die V. saphena magna zu schonen. Nach Spalten der oberflächlichen Faszie stellen sich die oberflächlichen Flexoren dar (Abb. 3a), welche Stumpf von der Tibia abgelöst werden können, um die tiefen Flexoren darzustellen, welche so ebenfalls eröffnet werden können (Abb. 3b).

**Lateralseitig**

Lateralseitig

sollte ca. 2 cm ventral der Fibula inzidiert werden. Danach wird das Subkutangewebe bis zur Faszie präpariert und das **Septum intermusculare**

**Figure 4:**
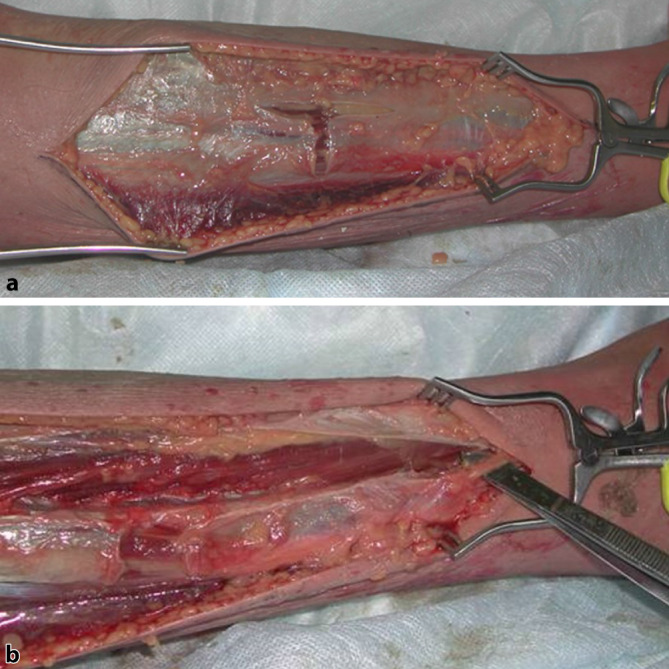

Septum intermusculare

dargestellt (Abb. 4a). Hierbei ist der **N. peroneus superficialis**

N. peroneus superficialis

zu schonen, welcher die Faszie distal durchtritt. Das anteriore und das laterale Kompartiment können nun problemlos gespalten werden (Abb. 4b).

**Figure 5:**
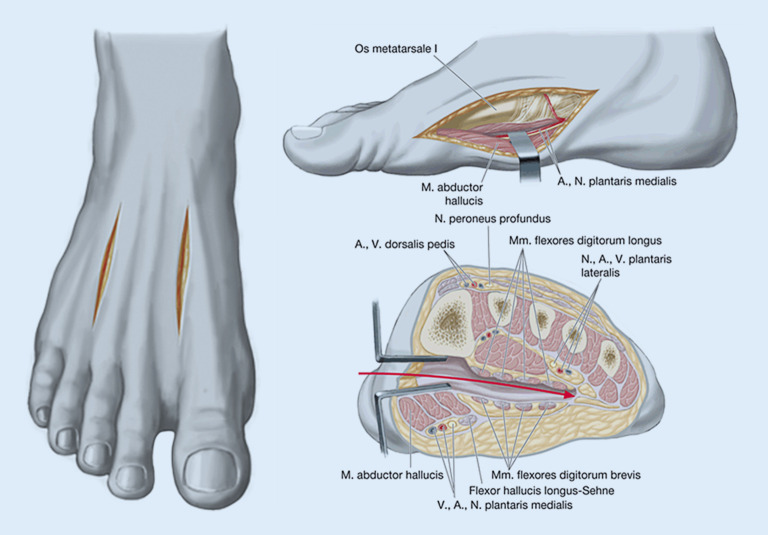

Der Vollständigkeit halber ist in den Abb. 5 und 6a, b noch eine schematische Darstellung der Zugangswege für die Kompartmentspaltung an Fuß sowie Ober- und Unterarm abgebildet [25, 26].

**Figure 6:**
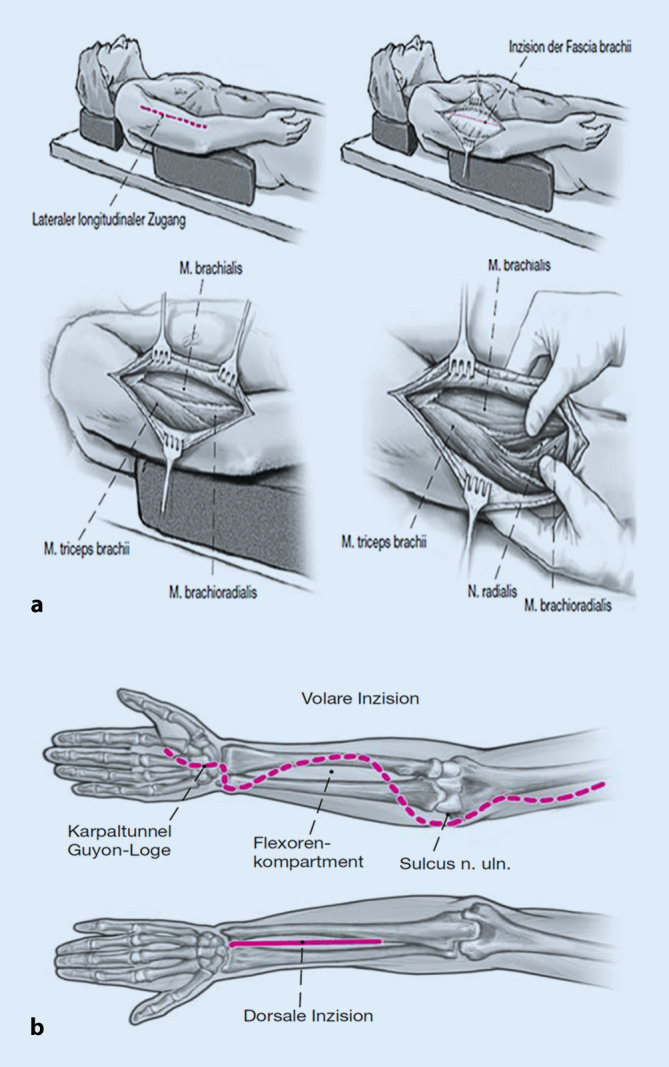

### Cave

*Kontraindikation zur operativen Therapie:* Ein akutes Kompartmentsyndrom, welches für länger als 24 h nicht gespalten wurde, sollte aufgrund des schlechten funktionellen Outcomes und des hohen Infektionsrisikos des bereits nekrotischen Gewebes nicht mehr gespalten werden. In diesem Fall sollten die Kompartimente verschlossen bleiben.

### Cave

*Kompartmentsyndrom beim Kind:* Das akute Kompartmentsyndrom beim Kind ist häufig und insbesondere beim Kleinkind erschwert zu diagnostizieren [27]. Bei einem passenden Unfallmechanismus und Verletzungsmuster sollte unbedingt daran gedacht und im Zweifelsfall eine erweiternde Diagnostik eingeleitet werden.

## Fazit für die Praxis

- Die notfallmäßige Dermatofasziotomie ist die einzige Behandlungsmöglichkeit beim akuten Kompartmentsyndrom der Extremität.
- Die Diagnose und Operationsindikation sollten schnellstmöglich gestellt werden, um potenziell tödliche Komplikationen zu vermeiden.
- Die häufigste Inzidenz liegt bei jungen, männlichen Patienten mit Hochrasanztraumata vor.
- Die am häufigsten betroffene Extremität ist der Unterschenkel, gefolgt von Vorderarm und Fuß.
- Beim bewusstseinsklaren Patienten sollte die Diagnose anhand der klinischen Untersuchung gestellt werden (das wichtigste Symptom ist hierbei unverhältnismäßig starker und analgetikaresistenter Schmerz).
- Eine Logendruckmessung kann bei uneindeutiger klinischer Untersuchung die Diagnosestellung unterstützen.
- Bei einem drohenden Kompartmentsyndrom sollte die Extremität auf Herzniveau gelagert und regelmäßig reevaluiert werden. Einengende Gipse und elastische, schnürende Verbände müssen umgehend entfernt werden.
- Bei der Dermatofasziotomie sollten lange, erweiterbare Schnitte vorgenommen und alle Kompartimente gespalten werden.

## CME-Fragebogen

Das akute Kompartmentsyndrom der Extremität …

ist eine seltene Komplikation, die meistens ohne kritische Folgen verläuft.

ist eine sehr seltene Komplikation mit potenziell tödlichem Verlauf.

ist eine häufige, schwere Komplikation bei Frakturen oder Weichteilverletzungen.

ist eine häufige Komplikation ohne größere klinische Relevanz.

kommt ausschließlich an der unteren Extremität vor.

Bei welchem dieser Patienten würden Sie am ehesten ein Kompartmentsyndrom vermuten?

Ein 20-jähriger Mann mit Unterschenkelfraktur nach Verkehrsunfall

Eine 80-jährige Frau mit einer distalen Radiusfraktur nach einem Stolpersturz

Ein 60-jähriger Mann mit einer Sprunggelenksfraktur nach einem Fehltritt beim Wandern

Ein 35-jähriger Mann mit einer offenen Oberarmfraktur nach tätlicher Auseinandersetzung

Eine 40-jährige Frau mit einem Weichteiltrauma am Oberschenkel nach Treppensturz

Welches dieser Symptome gehört *nicht* zu den klassischen Zeichen eines akuten Kompartmentsyndroms der Extremität?

Harte, gespannte Muskellogen

Starke, opiatresistente Schmerzen

Passiver Dehnungsschmerz

Sensomotorische Ausfälle

Fehlende periphere Pulse

Welche dieser möglichen Ursachen für ein Kompartmentsyndrom bewirkt eine Volumenabnahme der Kompartimente?

Eine atraumatische Blutung

Eine großflächige Verbrennung

Ein Paravasat nach Fehlinfusion

Ein ausgedehnter Weichteiltumor

Kapilläre Leckage bei nephrotischem Syndrom

Bei einem ansonsten gesunden Patienten mit eindeutiger klinischer Diagnose eines akuten Kompartmentsyndroms bei Unterschenkelfraktur sollte man …

das Bein hochlagern und in 2 h erneut klinisch evaluieren.

eine Logendruckmessung durchführen, um die Diagnose zu sichern.

die Fraktur mit einem zirkulären Gips schienen.

vor einer Intervention die Ergebnisse der Laboranalyse (Kreatinkinase, Myoglobin) abwarten.

eine notfallmäßige Dermatofasziotomie durchführen.

Sie führen eine Dermatofasziotomie am Unterschenkel durch. Hierbei sollten Sie …

falls möglich, nur das eindeutig betroffene Kompartiment entlasten.

eine Fraktur in jedem Fall primär mittels Plattenosteosynthese mitversorgen.

alle Logen mit ausgiebigen Schnitten entlasten.

die Schnittführung so sparsam wie möglich wählen.

nach der Faszienspaltung die Haut regelhaft primär verschließen.

In welchem dieser Fälle ist eine Dermatofasziotomie *kontraindiziert*?

Bei einer 70-jährigen Frau mit Kompartmentsyndrom am Unterschenkel unter NOAK (neue orale Antikoagulanzien) bei Vorhofflimmern

Bei einem 30-jährigen Mann mit hämorrhagischem Schock bei Polytrauma mit Kompartmentsyndrom am Unterarm

Bei einer 40-jährigen Frau mit i.v. Drogenabusus mit Kompartmentsyndrom am Unterarm

Bei einem 70-jährigen Mann mit verpasstem manifestem Kompartmentsyndrom des Unterschenkels seit 24 h

Bei einem 2 Jahre alten Mädchen mit Kompartmentsyndrom am Fuß nach Quetschtrauma

Welche dieser Komplikationen ist bei einem verpassten Kompartmentsyndrom der Extremität am *unwahrscheinlichsten*?

Amputation der Extremität bei ausgedehnter Nekrose

Akutes Nierenversagen bei ausgedehnter Rhabdomyolyse

Reanimationspflichtigkeit bei Reperfusionssyndrom

Fettemboliesyndrom

Funktionsverlust durch Nervenschädigung

Welche Aussage zur Logendruckmessung ist *inkorrekt*?

Die Logendruckmessung kann einmalig, wiederholt und kontinuierlich durchgeführt werden.

Die Differenz aus Diastole und Logendruck sollte 30 mm Hg nicht überschreiten.

Der abgelesene Wert beschreibt den absoluten Kompartmentdruck.

Bei einem klinisch gesicherten Kompartmentsyndrom sollte keine Logendruckmessung durchgeführt werden.

Die Logendruckmessung sollte bei liegendem Patienten mit Extremität auf Herzhöhe durchgeführt werden.

Sie haben einen 30-jährigen Patienten nach einem Verkehrsunfall in der Notaufnahme, der Schmerzen im linken Unterschenkel angibt, die mit Analgetika erträglich sind. Radiologisch kann eine Fraktur ausgeschlossen werden. Der Unterschenkel ist geschwollen und gespannt. Der Patient hat keine sensomotorischen Ausfälle. Was ist Ihr nächster Schritt?

Sie planen einen Kontrolltermin in 2 Tagen mit Ultraschalluntersuchung.

Sie schicken den Patienten mit Analgesie nach Hause, da er keine Fraktur hat.

Sie planen eine sofortige notfallmäßige Dermatofasziotomie.

Sie wickeln den Unterschenkel kompressiv und planen eine Magnetresonanztomographie für den Folgetag.

Sie nehmen den Patienten zur regelmäßigen klinischen Überwachung stationär auf und verabreichen Analgetika.

## References

[1] von Volkman R (1881). Die ischaemischen Muskellaehmungen und Kontrakturen. Zentralbl Chir.

[2] Ellis H (1958). Disabilities after tibial shaft fractures; with special reference to Volkmann’s ischaemic contracture. J Bone Joint Surg Br.

[3] Seddon HJ (1966). Volkmann’s ischaemia in the lower limb. J Bone Joint Surg Br.

[4] Matsen FA, Clawson DK (1975). The deep posterior compartmental syndrome of the leg. J Bone Joint Surg Am.

[5] Matsen FA, Winquist RA, Krugmire RB (1980). Diagnosis and management of compartmental syndromes. J Bone Joint Surg Am.

[6] Matsen FA (1983). A practical approach to compartmental syndromes. Part I. Definition, theory, and pathogenesis. Instr Course Lect.

[7] von Keudell AG (2015). Diagnosis and treatment of acute extremity compartment syndrome. Lancet.

[8] Sellei RM, Hildebrand F, Pape HC (2014). Acute extremity compartment syndrome: current concepts in diagnostics and therapy. Unfallchirurg.

[9] Hargens AR (1979). Peripheral nerve-conduction block by high muscle-compartment pressure. J Bone Joint Surg Am.

[10] Rorabeck CH, Clarke KM (1978). The pathophysiology of the anterior tibial compartment syndrome: an experimental investigation. J Trauma.

[11] Huard J, Li Y, Fu FH (2002). Muscle injuries and repair: current trends in research. J Bone Joint Surg Am.

[12] McQueen MM, Gaston P, Court-Brown CM (2000). Acute compartment syndrome. Who is at risk?. J Bone Joint Surg Br.

[13] Schwartz JT (1989). Acute compartment syndrome of the thigh. A spectrum of injury. J Bone Joint Surg Am.

[14] Halvachizadeh S, Jensen KO, Pape HC, Mauffrey C, Hak DJ, Martin IM (2019). Compartment syndrome due to patient positioning. Compartment syndrome: a guide to diagnosis and management.

[15] Kleshinski J (2008). Review of compartment syndrome due to group A streptococcal infection. Am J Med Sci.

[16] Schmidt AH (2017). Predicting acute compartment syndrome (PACS): the role of continuous monitoring. J Orthop Trauma.

[17] Rameder P (2019). Epidemiology, treatment and outcome after compartment syndrome of the thigh in 69 cases—Experiences from a level I trauma centre. Injury.

[18] Richter M (2001). Foot fractures in restrained front seat car occupants: a long-term study over twenty-three years. J Orthop Trauma.

[19] Jeffers RF (2004). Prevalence and patterns of foot injuries following motorcycle trauma. J Orthop Trauma.

[20] McQueen M (1998). Acute compartment syndrome. Acta Chir Belg.

[21] Moehring HD, Voigtlander JP (1995). Compartment pressure monitoring during intramedullary fixation of tibial fractures. Orthopedics.

[22] Ulmer T (2002). The clinical diagnosis of compartment syndrome of the lower leg: are clinical findings predictive of the disorder?. J Orthop Trauma.

[23] Sheridan GW, Matsen FA (1976). Fasciotomy in the treatment of the acute compartment syndrome. J Bone Joint Surg Am.

[24] Jäger C, Zeichen J (2011). Das akute Kompartmentsyndrom des Unterschenkels. Orthop Traumatol.

[25] Zwipp H, Rammelt S (2014). Komplextrauma und plastische Rekonstruktion. Tscherne Unfallchirurgie: Fuß.

[26] Mittlmeier T, Krapohl BD, Schaser KD (2010). Management of severe soft-tissue trauma in the upper extremity—shoulder, upper and lower arm. Oper Orthop Traumatol.

[27] Mortensen SJ (2020). Risk factors for developing acute compartment syndrome in the pediatric population: a systematic review and meta-analysis. Eur J Orthop Surg Traumatol.

